# MicroRNA-425-5p modulates osteoporosis by targeting annexin A2

**DOI:** 10.1186/s12979-021-00256-7

**Published:** 2021-12-08

**Authors:** Guanghua Chen, Guizhi Huang, Han Lin, Xinyou Wu, Xiaoyan Tan, Zhoutao Chen

**Affiliations:** Department of Orthopedics, Affiliated Hospital of Guangdong Medical University, NO.57, Renmin Dadao, Xiashan District, Zhanjiang, 524001 Guangdong Province China

**Keywords:** miRNA-425-5p;osteoporosis;osteogenic differentiation;ANXA2

## Abstract

**Background:**

Studies have shown that the decrease of osteogenic differentiation of bone marrow mesenchymal stem cells (MSC) is an important mechanism of osteoporosis. The object of this study was to explore the role and mechanism of microRNA miR-425-5p in the differentiation of MSC.

**Methods:**

The expression of miR-425-5p in MSC was detected by quantitative reverse transcriptase-polymerase chain reaction (qRT-PCR). Cell proliferation, cell cycle and apoptosis were detected by CCK-8 colorimetry and flow cytometry. The expression of TNF were detected by ELISA.

**Results:**

Our data show that MiR-425-5p could modulate TNF-induced cell apoptosis, proliferation, and differentiation. ANXA2 is also the target of miR-425-5p and ANXA2 was involved in TNF-induced MSC cell apoptosis, proliferation, and differentiation. In addition, MiR-425-5p enhanced osteoporosis in mice.

**Conclusion:**

MiR-425-5p might serve as a potential therapeutic target for the treatment of osteoporosis.

## Introduction

Osteoporosis is a common metabolic bone disease characterized by inhibited bone mass, inhibited bone densities and bone microstructural destruction [1, 2]. The bone is prone to fragility fractures [3]. The incidence of osteoporotic fractures continues to increase, which will seriously jeopardize the quality of life of the elderly population [4]. The main cause of osteoporosis is the imbalance between bone resorption and bone formation [5]. Mesenchymal stem cells (MSC) are one type of stem cells derived from mesoderm [6]. They have multi-directional differentiation potential and exist in various tissues such as the liver, skin, and placenta [7]. Studies have found that the reduction of MSC to osteogenic differentiation is an important mechanism for the pathogenesis of osteoporosis [8]. Promotion of its osteogenic differentiation to correct bone metabolism imbalance is one of the directions for the treatments of osteoporosis [9]. Signaling pathways, microRNAs (miRNAs), and epigenetic modifications are all involved in the regulation of MSC differentiation and fate [10]. Identification of the key factors determining the differentiation direction of MSC transplantation will provide new insights into the treatment of osteoporosis [11].

Recent studies have demonstrated that miRNAs could regulate the expression of downstream genes to exert suppressive roles in many human diseases [12]. Previous studies have found that they play important roles in cell proliferation, differentiation, apoptosis, signal transduction and other life processes as a conservative and universal regulatory mechanism [13, 14]. Changes in the miRNAs expression profile of cells will have a great impact on the state and function of stem cells [15, 16]. Studies have found that a variety of miRNAs play important regulatory roles in the differentiation of MSC, such as miR-23b [17], miR-320 [18], and miR-9 [19]. MiR-425-5p has been shown to play an essential role in several human cancers. One study reported that miR-425-5p was greatly inhibited in acute myeloid leukemia [20]. However, little information has been revealed in MSC differentiation.

Annexin A2 (ANXA2) is a class of calcium-dependent membrane phospholipid-binding proteins involved in important life processes such as cell proliferation, differentiation, apoptosis and migration [21]. Studies have found that the ANXA2 gene is involved in the pathogenesis of osteoporosis [21, 22], but its mechanism in MSC differentiation has not been reported. Our preliminary bioinformatic analysis showed that miR-425-5p could bind with ANXA2. Therefore, we hypothesized that miR-425-5p might exert a role in MSC differentiation by interacting with ANXA2. This study was carry out to investigate the functions of miR-425-5p and ANXA2 in MSC differentiation. Our findings would provide a new therapeutic target for the treatment of osteoporosis.

## Materials and methods

### Animals

Female C57BL/6 J mice (*n* = 33) aged at 35 days and weighted ~ 18 g were provided by Vital River Laboratory, China. Mice were maintained at ~ 23 °C with a 12 h/12 h day/night light cycle. The mice had free access to food or drink. This study was approved by the Animal Care Committee of Affiliated Hospital of Guangdong Medical University.

### Cell culture

MSC purified from the marrow of 3 healthy mice (female, C57BL/6 J) were incubated in a-minimum essential medium supplemented with 10% FBS, 2 mM L-glutamine, 100 U/ml penicillin, and 100 mg/ml streptomycin at 37 °C with 5% CO_2_. Cells were incubated in MSC growth medium with 10 nM dexamethasone, 20 mM β-glycerol phosphate, and 50 μM L-ascorbic acid 2-phosphate for 1 week to generate osteogenic differentiation of MSC. For the osteogenic differentiation of MSCs, cells were cultured in MSCs growth medium supplemented with 10 nM dexamethasone, 20 mM b-glycerol phosphate, and 50 lM L-ascorbic acid 2-phosphate for 7 d.

### Transfection

MiR-425-5p mimics and NC-NC-mimics (100 pmol, Genepharma, China) were transfected to MSC with Lipofectamine 2000 (Invitrogen, USA). Then, si-ANXA2, si-control, ANXA2 or vector (Nanjing Kaiji Biotech, China) were transfected to MSC with Lipofectamine 2000.

### qRT-PCRs

Total RNAs were extracted from MSC using TRIzol (Invitrogen). Reverse transcription was performed using the PrimeScript kit (Takara, Japan). qRT-PCRs were conducted on an ABI Prism 7500 system (Applied Bio, USA). SYBR Assays (Qiagen, CA) were conducted to measure the expression levels of ANXA2 and β-actin. TaqMan kit (Applied Bio., USA) was used to measure the expression levels of miR-425-5p. U6 and GAPDH were used as the endogenous control for miR-340-5p and STAT3, respectively. Gene expression levels were quantified by the 2^−ΔΔCt^ method. The primer sequences were listed in Table 1.

**Table 1:** Sequences of primers used in qRT-PCRs

Gene	Forward primer (5′ - 3′)	Attenuated primer (5′ - 3′)
miR-425-5p	TGCGGAATGACACGATCACTCCCG	CCAGTGCAGGGTCCGAGGT
U6	CTCGCTTCGGCAGCACA	AACGCTTCACGAATTTGCGT
ANXA2	GTGGTGGAGATGACTGAAGCC	CCACGGGGACTGTTATTCG
β-actin	ATTGCCGACAGGATGCAGAA	CAAGATCATTGCTCCTCCTGAGCGCA

### Western blotting

MSC was lysed by RIPA lysis buffer. Proteins were separated by 12% SDS-PAGE and transferred to nitrocellulose membranes. After incubation in 5% skim milk for 1 h, the membrane was incubated with anti-osteocalcin, anti-osterix, anti-ANXA2, and anti- β-actin at 1:1000 (Santa Cruz Biotech, USA) at 4 °C for overnight. The membrane was then washed and incubated with HRP-conjugated secondary antibody at 1:5000 (Sigma, USA) at 25 °C for 1 h. ECL kit (Pierce Chemical, USA) was used to visualize the blots.

### Apoptotic cells

MSC collected at 2 d post-transfection was cleaned and suspended in 500 μl binding buffer. MSC was then incubated with Annexin V at 25 °C for 10 min and stained by propidium iodide. Flow cytometry assay was performed to analyze the results.

### Cell proliferation

Bromodeoxyuridine (BrdU) kit (Millipore, USA) was utilized to quantify the proliferation of MSC at 2 d post-transfection. Cells proliferation was detected at 450 nm using a microplate reader (Bio-Rad, USA).

### Luciferase activity assay

The 3′-UTR of ANXA2 with the binding sites of miR-425-5p or mutants were inserted to pmirGLO miRNA vector (Promega, USA). The recombinant constructs were co-transfected with miR-425-5p mimics or NC to MSC and incubated for 2 d. Luciferase activities were quantified by Dual-Luciferase assays.

### Osteoporosis model in mice

The mice were randomly grouped into 5 groups: the blank control (Control), sham operation (Sham), ovariectomized (osteoporosis model), osteoporosis model with miR-425-5p mimics (osteoporosis model + miR-425-5p mimics) and osteoporosis model with NC-mimics (osteoporosis model + NC-mimics). Mice were subcutaneously anesthetized by 3% sodium pentobarbital (30 mg/kg body weight, Sigma Chemical Co., St. Louis, MO, USA) and had bilateral laparotomy (Sham) or bilateral oophorectomy (osteoporosis model). Then, 0.02 mg/kg fentanyl citrate (Abbott, USA) was injected subcutaneously 2 times per day for 3 ds after surgeries. NC-mimics or miR-425-5p mimics (0.5 nmol in 1 μl in vivo transfection reagent) (Entranster, China) was delivered by subcutaneous injection (osteoporosis model + miR-425-5p mimics or osteoporosis model + NC-mimics) once daily for 3 d after surgery. For euthanasia, mice were deeply anesthetized with 3% sodium pentobarbital (30 mg/kg body weight) through intraperitoneal injection and sacrificed by cervical dislocation.

### Detection of serums parameters

Mice were anesthetized 3 months after the construction of osteoporosis model. Blood was collected, clotted, and centrifuged. Serum samples were collected and the osteocalcin concentration was quantified by ELISA (Rapidbio, USA).

### Bone mineral densities measurements

Dual-energy X-ray absorptiometry (DEXA) (Lunar, USA) was used to measure bone mineral densities of the right femur mid-diaphysis. Bone mineral density (BMD) was calculated by bone mineral contents (g) and bone areas (cm^2^) as g/cm^2^.

### ELISA (serum alkaline phosphatase measurements)

Serum ALP is mainly used for the diagnosis and differential diagnosis of skeletal and hepatobiliary diseases. Collected mice serum samples, let them stand for 2 h, and then centrifuged the samples to get the supernatant. Then we used the mice alkaline phosphatase (ALP) detection kit to operate step by step according to the steps in the manual, microplate reader was used to read the absorbance 450 nm, calculate statistics, and detect each sample for three times.

### Statistical analysis

Data analyses were conducted using SPSS 19.0 software. Data from 3 independent replicates were expressed as mean ± standard deviation (SD) values. Differences among various groups were compared by unpaired t-test or one-way ANOVA. *P* < 0.05 was regarded as significant difference.

## Results

### MiR-425-5p could modulate TNF-induced cell apoptosis, proliferation, and differentiation

**Fig. 1:**
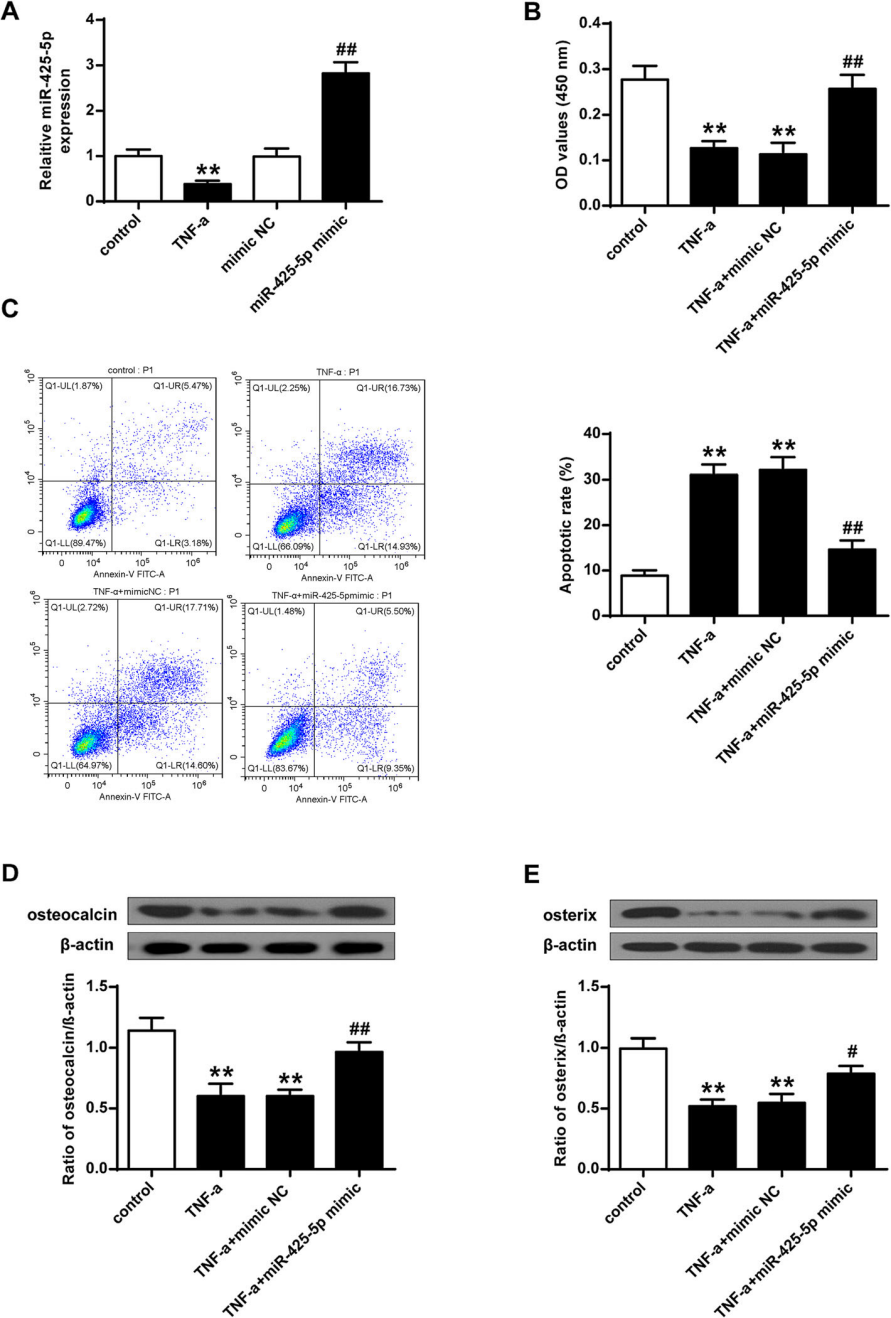
MiR-425-5p could modulate TNF-related cell apoptosis, proliferation, and differentiation. **A** The expression levels of miR-425-5p were quantified by qRT-PCRs. **B** Cell proliferation was quantified by BrdU assays. **C** Cell apoptosis was quantified by flow cytometry. **D** The expression levels of osteocalcin and osterix were quantified by western blotting. **E** Densitometric analysis of the results from. β-actin was utilized as an internal control. Data from 3 independent replicates were expressed as mean ± standard deviation (SD) values.*n* = 3, **P < 0.01 vs control; # *P* < 0.05, ## *P* < 0.01 vs NC-mimics or TNF-α + NC-mimics

To investigate the role of miR-425-5p, the expression of miR-425-5p was firstly evaluated in TNF treated MSCs. As shown in Fig. 1A, the expression levels of miR-425-5p in TNF treated MSCs were significantly reduced (*P* < 0.01). In addition, cell proliferation was greatly suppressed by TNF in MSCs (*P* < 0.01), and over-expression of miR-425-5p attenuated the effect of TNF on MSC proliferation (*P* < 0.01) (Fig. 1B). After transfecting the miR-425-5p mimics into MSCs, the expression levels of miR-425-5p were significantly increased (*P* < 0.01). As shown in Fig. 1C, the apoptotic cells of MSCs were greatly induced by TNF (*P* < 0.01), which were suppressed by miR-425-5p mimics (*P* < 0.01). Furthermore, the expression of osteogenic markers, osteocalcin and osterix were suppressed by TNF treatments (*P* < 0.01). The expression levels of osteocalcin and osterix elevated after the over-expression of miR-425-5p (*P* < 0.05; *P* < 0.01) (Fig. 1D and E). These results demonstrated that miR-425-5p may modulate cell apoptosis, proliferation, and differentiation of TNF-induced MSC.

### ANXA2 was targeted by miR-425-5p in MSCs

**Fig. 2:**
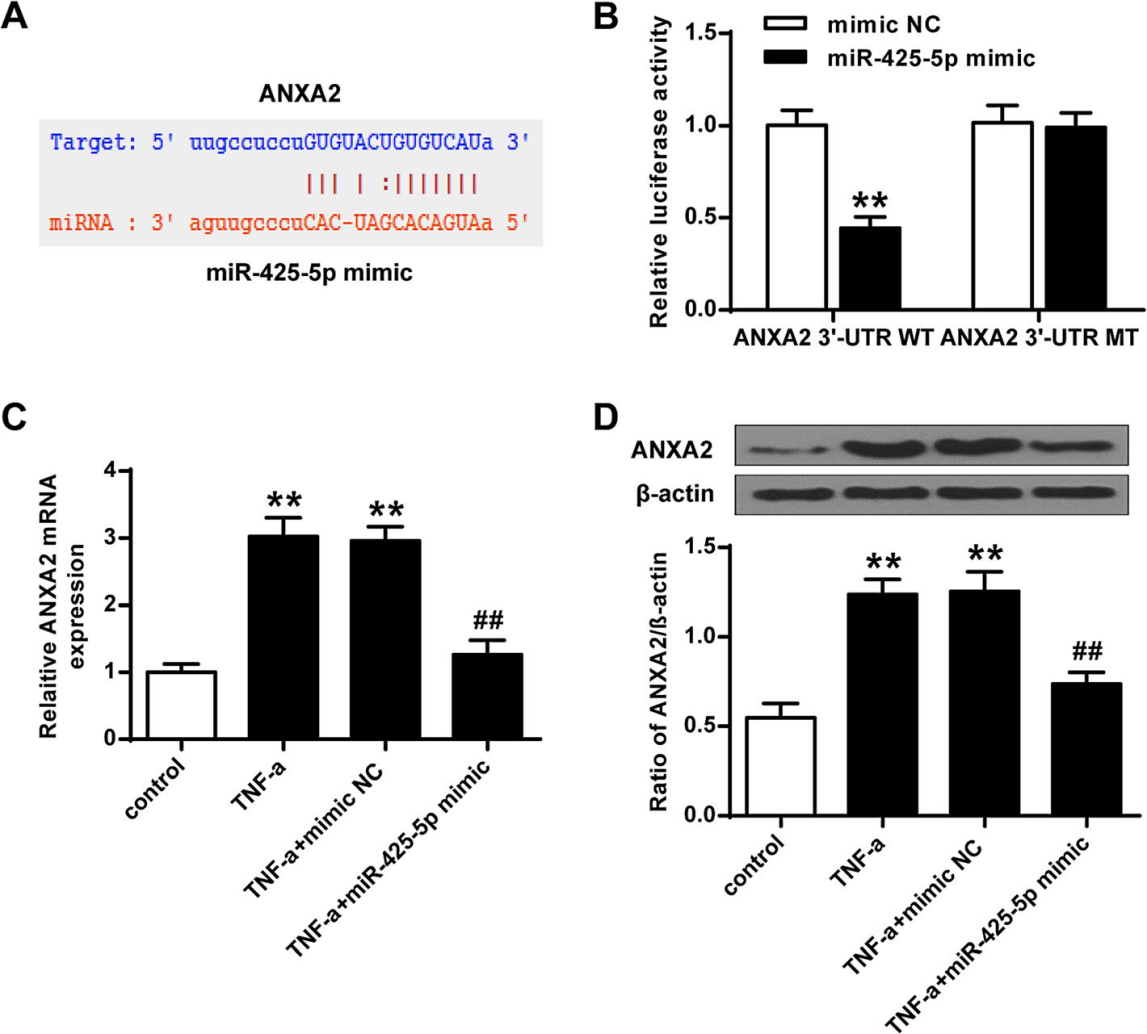
ANXA2 was targeted by miR-425-5p. **A** Shared common sequences between miR-425-5p and ANXA2 3′-UTR. **B** Luciferase activities with various treatments. **C** mRNA level of ANXA2 in MSC with various treatments. **D** The protein level of ANXA2 in MSC with various treatments. Data from 3 independent replicates were expressed as mean ± standard deviation (SD) values.*n* = 3, **P < 0.01 vs NC-mimics or control group; ## *P* < 0.01

To investigate the underlying mechanism of miR-425-5p in regulating osteoporosis, the functional target gene of miR-425-5p was identified. Bioinformatics analysis results showed that ANXA2 was targeted by miR-425-5p (Fig. 2A). In addition, over-expression of miR-425-5p greatly inhibited the luciferase activities of the wild type ANXA2 3′-UTR (*P* < 0.01), but had no impact on the luciferase activities of the reporter vector containing the mutant 3′-UTR of ANXA2 (*P* > 0.05) (Fig. 2B). Furthermore, over-expression of miR-425-5p greatly inhibited the expression of ANXA2 (*P* < 0.01) (Fig. 2C and D). These results suggested that ANXA2 was targeted by miR-425-5p.

### ANXA2 was involved in TNF-induced MSC cell apoptosis, proliferation, and differentiation

**Fig. 3:**
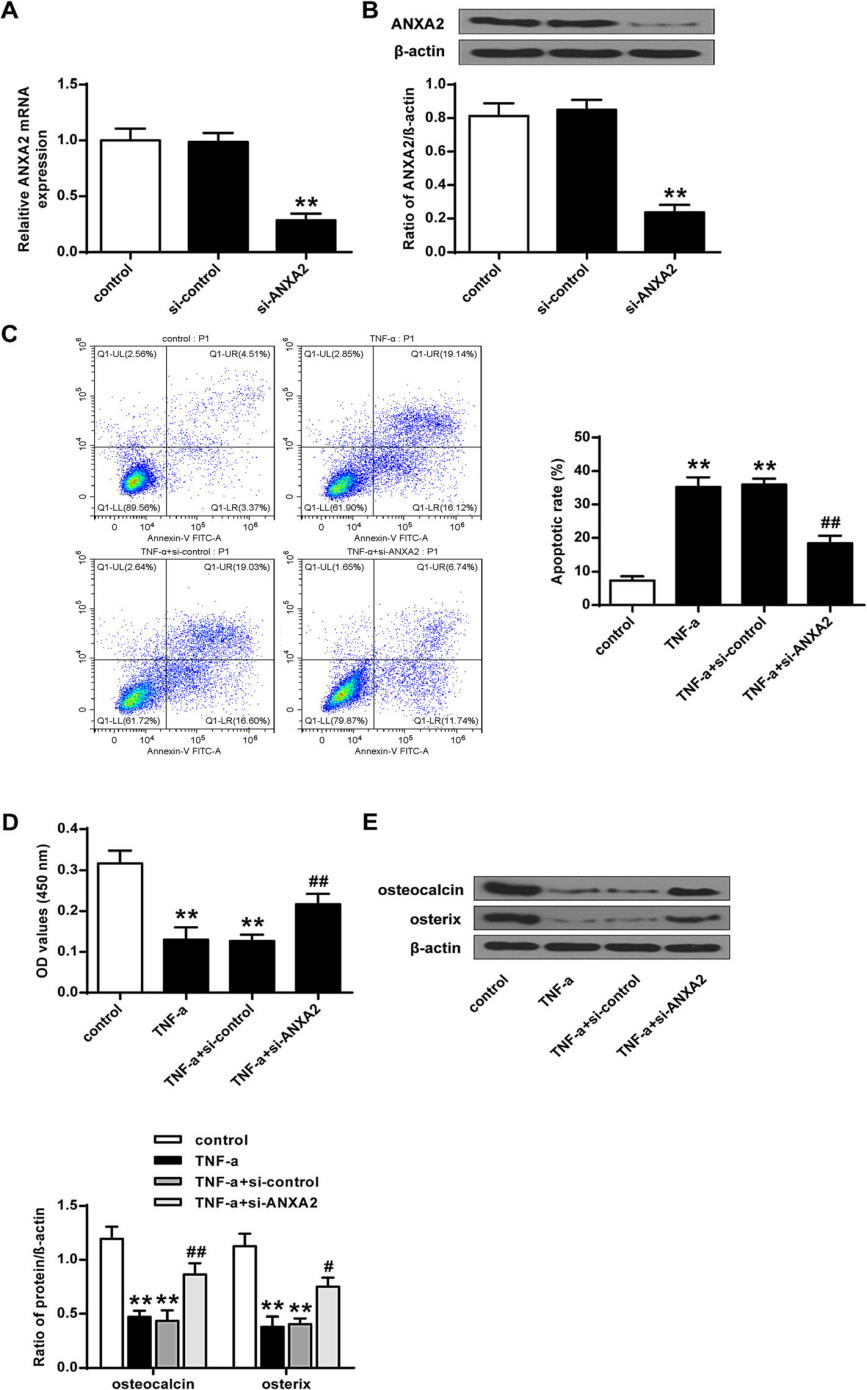
Knockdown of ANXA2 modulated TNF-related cell apoptosis, proliferation, and differentiation. **A** The mRNA level of ANXA2 in MSC with various treatments. **B** The protein level of ANXA2 in MSC with various treatments. **C** apoptotic cells of MSC with various treatments. **D** Cell proliferation of MSC with various treatments. **E** The Expression of osteocalcin and osterix in MSC with various treatments. *n* = 3, **P < 0.01 vs control or si-control; ##*P* < 0.01 vs TNF-α + si-control vs TNF-α + NC-mimics

As shown in Fig. 3A and B, the expression of ANXA2 was greatly inhibited by knockdown of ANXA2 at both mRNA and protein levels (*P* < 0.01). In addition, knockdown of ANXA2 attenuated the effect of TNF on MSC cell apoptosis, proliferation, and differentiation (*P* < 0.05, *P* < 0.01) (Fig. 3C-E). These results suggested that knockdown of ANXA2 could modulate TNF-induced cell apoptosis, proliferation, and differentiation.

### Over-expression of ANXA2 attenuated the effect of miR-425-5p on TNF treated MSC function

**Fig. 4:**
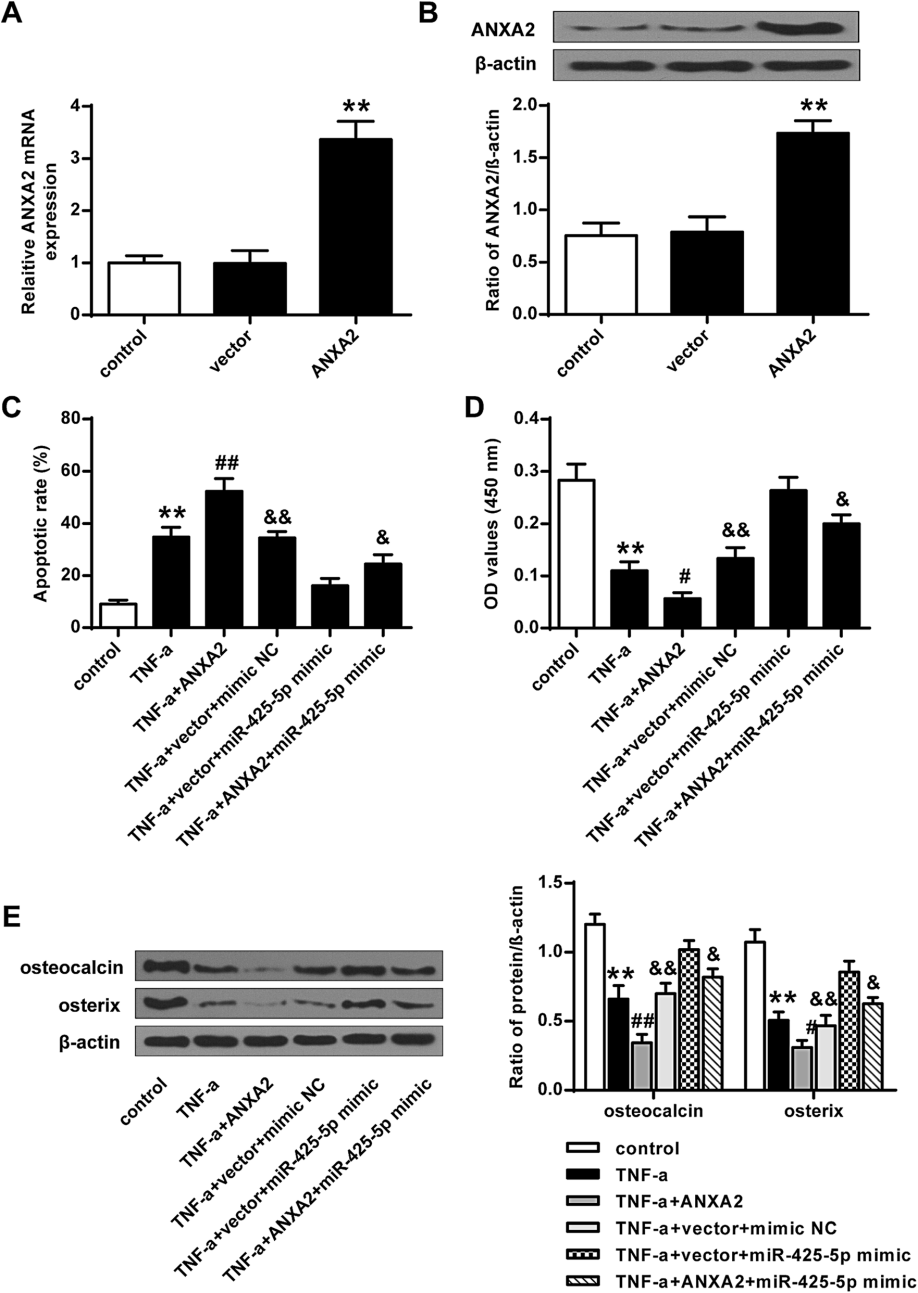
Over-expression of ANXA2 attenuated the impact of miR-425-5p. **A** The mRNA level of ANXA2 in MSC with various treatments. **B** The protein level of ANXA2 in MSC with various treatments. **C** apoptotic cells of MSC with various treatments. **D** Cell proliferation of MSC with various treatments. **E** The expression of osteocalcin and osterix in MSC with various treatments. Data from 3 independent replicates were expressed as mean ± standard deviation (SD) values.*n* = 3,*P < 0.05, **P < 0.01 vs control or vector group; #*P* < 0.05, ##*P* < 0.01 vs TNF-α group; &*P* < 0.05, &&*P* < 0.01 vs TNF-α + vector + miR-425-5p mimics group

As shown in Fig. 4A and B, the expression levels of ANXA2 were greatly elevated by over-expression of ANXA2 at both mRNA and protein levels (*P* < 0.01). Flow cytometry assay (Fig. 4C), BrdU assay (Fig. 4D) and Western blotting analysis (Fig. 4E) all demonstrated that over-expression of ANXA2 could greatly attenuate the effect of miR-425-5p on TNF treated MSCs (*P* < 0.01).

### MiR-425-5p enhanced osteoporosis in mice

**Fig. 5:**
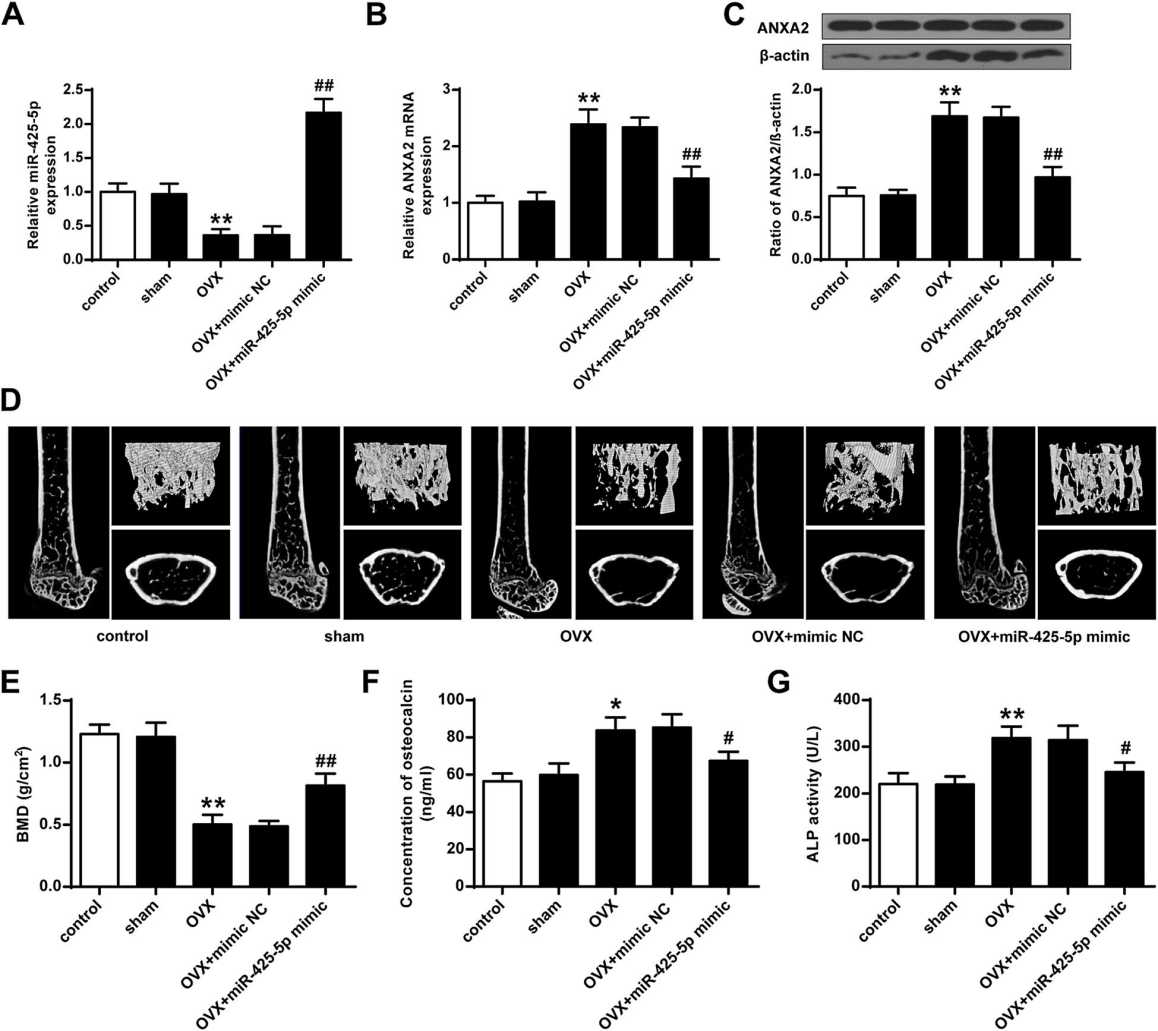
miR-425-5p enhanced osteoporosis in mice. **A** miR-425-5p level in the MSC. **B** The protein level of ANXA2 in MSC. **C** protein level of MT1-MMP in MSC. **D** BMD level of femurs. **E** The concentration of osteocalcin in serums. **F** ALP activities in serums. Data from 3 independent replicates were expressed as mean ± standard deviation (SD) values.*n* = 3,*P < 0.05, **P < 0.01 vs control or sham; # *P* < 0.05, ## *P* < 0.01 vs osteoporosis model + NC-mimics

The expression levels of miR-425-5p were greatly reduced in osteoporosis model mice compared to that in the control mice (*P* < 0.01) (Fig. 5A). The expression levels of ANXA2 were elevated in the osteoporosis model mice at both mRNA and protein levels (*P* < 0.01), while reduced in osteoporosis model mice transfected with miR-425-5p mimics (*P* < 0.01) (Figs. 5B and C). In addition, the BMD of the osteoporosis model mice was greatly inhibited compared with the mice in the Sham group (*P* < 0.01), and transfection with miR-425-5p mimics enhanced the BMD of osteoporosis model mice (*P* < 0.01) (Fig. 5D and E). Furthermore, the osteocalcin level and ALP activities in serum were elevated in the osteoporosis model mice (*P* < 0.05, *P* < 0.01), and were inhibited in the osteoporosis model mice with the transfection of miR-425-5p mimics (*P* < 0.01) (Fig. 5F and G). These results indicated that miR-425-5p could enhance osteoporosis in mice.

## Discussion

TNF is a potent inducer of bone resorption [23]. It was reported that human TNF gene-transfection could enhance bone resorption of osteoclasts in nude mice [24]. Therefore, we treated the MSCs with TNF to induce the MSC apoptosis and to investigate the functions of miR-425-5p in MSCs [25]. We found that the expression of miR-425-5p was greatly suppressed in MSC. Apoptotic cells of MSC were greatly induced by TNF but were suppressed by over-expression of miR-425-5p. Cell proliferation was greatly suppressed by TNF in MSC, and over-expression of miR-425-5p attenuated the down-regulation effect. The expression of osteogenic markers, osteocalcin, and osterix were suppressed by TNF treatments. In addition, the expression levels of osteocalcin and osterix were elevated after the over-expression of miR-425-5p. Previous studies have demonstrated that various miRNAs could modulate the differentiation and apoptosis of MSC, such as miR-23b [17], miR-574-3p [26], and miR-320 [18]. For the first time, we report that miR-425-5p could modulate TNF-related cell apoptosis, proliferation, and differentiation.

Previous studies have shown that ANXA2 could modulate MSC differentiation by interacting with several specific miRNAs such as miR-9 [27], miR-155 [28], and miR-206 [29]. We found that ANXA2 was targeted by miR-425-5p. Therefore, we hypothesized that miR-425-5p may interact with ANXA2 to modulate MSC differentiation. In this study, over-expression of miR-425-5p greatly inhibited the luciferase activities of the wild type ANXA2 3′-UTR, but had no obvious impact on the mutant ANXA2 3′-UTR. Over-expression of miR-425-5p greatly inhibited the expression of ANXA2. To the best of our knowledge, we are the first to report that ANXA2 was targeted by miR-425-5p.

TNF could induce the cell apoptosis of MSC. It was reported that ANXA2 attenuated osteoblast growth and is associated with hip BMD and osteoporotic fracture [22]. From our study, it was found that knockdown of ANXA2 attenuated the impact of TNF on cell apoptosis, proliferation, and differentiation in MSC cells. In addition, knockdown of ANXA2 modulated TNF-related cell apoptosis, proliferation, and differentiation. Flow cytometry assay, BrdU assay, and western blotting analysis demonstrated that over-expression of ANXA2 could greatly attenuate the impact of miR-425-5p on TNF treated MSC. Also, over-expression of ANXA2 attenuated the impact of miR-425-5p on MSC differentiation.

The roles of miR-425-5p and ANXA2 in TNF induced MSC apoptosis were explored in vitro, and in vivo experiments could better support their inner associations [30]. The expression of miR-425-5p was greatly inhibited in osteoporosis model compared to that in the control mice. The expression levels of ANXA2 were elevated in the osteoporosis mice model mice but were inhibited in osteoporosis model mice with the over-expression of miR-425-5p. It is well known that the expression levels of plasma miRNAs are correlate with sensitivity to bone mineral density in postmenopausal osteoporosis patients [31]. The BMD of the osteoporosis model mice was greatly inhibited compared to mice in the Sham mice. Over-expression of miR-425-5p enhanced the BMD of osteoporosis model mice. The osteocalcin level and ALP activities in serums were elevated in the osteoporosis model mice and inhibited in the osteoporosis model mice with the over-expression of miR-425-5p. Our findings suggested that miR-425-5p could enhance osteoporosis in mice.

## Conclusion

In conclusion, miR-425-5p could modulate osteoporosis by targeting ANXA2 both in vitro and in vivo. Our findings would provide new insights into the diagnostic and prognostic of osteoporosis.

## Data Availability

The analyzed data sets generated during the study are available from the corresponding author on reasonable request.

## Abbreviations

MSC: Mesenchymal stem cells
miRNA: microRNA
